# Emotion‐Related Treatments in Patients With Binge Eating Episodes—a Systematic Review and Meta‐Analysis

**DOI:** 10.1111/obr.70058

**Published:** 2025-12-12

**Authors:** Kathrin Schag, Jessica Werthmann, Elisabeth Johanna Leehr, Hanna Preuss‐van‐Viersen, Eva‐Maria Skoda, Vanessa Rentrop, Fabian Breuer, Maarit Pelzer, Laura Derks, Florian Hammerle, Arne Bürger, Tanja Legenbauer

**Affiliations:** ^1^ Department of Psychosomatic Medicine and Psychotherapy University Hospital Tübingen Tübingen Germany; ^2^ Centre of Excellence for Eating Disorders Tübingen (KOMET) Tübingen Germany; ^3^ Clinical Psychology and Psychotherapy Unit, Institute of Psychology Albert‐Ludwigs University of Freiburg Freiburg Germany; ^4^ Institute for Translational Psychiatry University of Münster Münster Germany; ^5^ Department of Child and Adolescent Psychiatry and Psychotherapy University Medical Center of the Johannes Gutenberg University Mainz Mainz Germany; ^6^ Clinic for Psychosomatic Medicine and Psychotherapy, LVR‐University Hospital Essen University of Duisburg‐Essen Essen Germany; ^7^ Center for Translational Neuro‐ and Behavioural Sciences (C‐TNBS) University of Duisburg‐Essen Essen Germany; ^8^ LWL‐University Hospital for Child and Adolescent Psychiatry, Psychotherapy and Psychosomatics Ruhr University Bochum Hamm Germany; ^9^ Department of Child and Adolescent Psychiatry, Psychosomatics and Psychotherapy, Center of Mental Health University Hospital of Wuerzburg Wuerzburg Germany; ^10^ German Centre of Prevention Research in Mental Health University of Wuerzburg Würzburg Germany

**Keywords:** binge eating, eating disorder, emotion, psychotherapy

## Abstract

Emotions represent potential triggers for binge eating, and binge eating can serve as a dysfunctional emotion regulation strategy. Therefore, we investigated emotion‐related treatments in patients with binge eating in a systematic review. Change in binge eating were the primary outcome; eating disorder pathology and emotion‐related outcomes were secondary outcomes. A meta‐analysis was computed regarding pre‐changes and post‐changes in binge eating frequency within groups, and potential influencing factors were investigated, namely, BMI, eating disorder diagnosis, treatment type, aspects of BE outcome, age, and sex. Thirty‐eight articles were included and 31 within the meta‐analysis. Data quality was rated as moderate. The sample size varied strongly with a high proportion of women. Only five studies examined adolescents. Binge eating disorder (*n* = 18) and dialectical behavioral treatment (*n* = 16) were most frequently examined. Results show significant reductions in binge eating after treatment. Additionally, the systematic review indicates the superiority of emotion‐related treatments compared with waitlist control groups and comparability with active control groups in terms of improvements in binge eating, eating disorder pathology, and emotion regulation. The effects appear to be stable at follow‐up analyses from 1 to 12 months. Potential influencing factors did not affect the efficacy of emotion‐related treatments. Overall, though meta‐analytic results have to be interpreted with caution, emotion‐related treatments hold promise in treating binge eating, and emotion regulation might represent a potential mechanism of change.

**Abbreviations**
AN‐BP: anorexia nervosa binge‐purge type
BE: binge eating
BED: binge eating disorder
BES: Binge Eating Scale
BN: bulimia nervosa
CBT: cognitive behavioral treatment
DBT: dialectic behavioral therapy
DEBQ: Dutch Eating Behavior Questionnaire
DERS: Difficulties in Emotion Regulation Scale
ED: eating disorder
EDE: Eating Disorder Examination
EDE‐Q: Eating Disorder Examination Questionnaire
EDI: Eating Disorder Inventory
EES: Emotional Eating Scale
ER: emotion regulation
ET: emotion‐related treatment
EFT: emotion‐focused therapy
ICAT: integrative cognitive‐affective therapy
FU: follow‐up
LOC: loss of control eating
NMRS: Negative Mood Regulation Scale
OSFED: other specified eating disorders
PRISMA: Preferred Reporting Items for Systematic reviews and Meta‐Analyses
RCT: randomized controlled trial
SIAB: Structured Interview for Anorexia Nervosa and Bulimia Nervosa
UFED: unspecified eating disorders

## 1 Introduction

Binge eating (BE) behavior is highly associated with obesity. This is reflected in elevated prevalence rates of 30%–40% of obesity in patients with binge eating disorder (BED) and bulimia nervosa (BN) [1, 2]. Therefore, the reduction of BE is important to facilitate weight reduction, and eating disorder (ED) treatment is impeded by co‐occurring obesity [2, 3]. Like others, we support a transdiagnostic perspective on EDs with regular BE [4, 5, 6]. For example, Williamson and colleagues [6] conclude from their analyses that patients with BE, but different diagnoses, build one entity. Psychological treatment, particularly cognitive behavioral treatment (CBT), is the first‐line treatment for BE, especially in BED and BN [3, 7, 8, 9]. However, only 50% of patients with BED and 30% of patients with BN respond to psychological treatments with remission 1 year after treatment [7, 10, 11]. Therefore, it is necessary to identify effective mechanisms of change and to further advance treatment. A primary candidate to enhance treatment outcome is a training of emotion regulation (ER), because emotion dysregulation contributes to BE [2, 12]. There is substantial empirical evidence relating negative mood and BE, especially regarding sadness, anger, shame, and stress in BED [13, 14, 15]. Evidence shows that negative mood acts as a trigger for BE episodes [16, 17, 18], and BE itself may be understood as a maladaptive ER strategy to achieve relief from these negative emotions [16, 19, 20], as more adaptive strategies are lacking [21, 22, 23]. Additionally, some work indicates that after an immediate relief, negative emotions increase again in patients with BED due to feelings of guilt, shame, and self‐denial, so that a vicious circle unfolds [13, 14]. In addition to the maladaptive use of ER strategies, patients with BE show problems with emotional awareness; that is, they often fail to recognize emotions appropriately [23, 24]. Thus, enhancing emotional awareness and use of adaptive ER strategies could enhance the outcome of BE treatment. Accordingly, the aim of the current review is to investigate whether psychological treatments that are directly targeting emotions, that is, so‐called emotion‐related treatments (ET), are effective in the treatment of BE. To explore potential underlying mechanisms, we investigate not only change in BE but also change in ER as well as the relation between change in ER with BE and other treatment outcomes.

Unfortunately, there currently exists no conclusive definition of ET. Like others [25, 26], we therefore chose a bottom‐up approach to be as inclusive as possible and included all studies among individuals with BE in this review stating that the used psychological treatment is directly targeting emotions, for example, where core interventions address the implementation of emotional awareness or adaptive ER strategies. For example, we included third‐wave therapies, where ER is treated with strategies of acceptance and mindfulness [27]. In particular, dialectical behavioral therapy (DBT) [28, 29, 30], mindfulness‐based therapies, and acceptance and commitment therapy [31, 32, 33] show promising results regarding treatment of BE. Additionally, there are several other psychological treatments targeting emotions that are included in this review as well, for example, emotion‐focused therapy (EFT) [34] or integrative cognitive‐affective therapy (ICAT) [35]. Other treatments that address emotions in a more indirect way like classical CBT or interpersonal psychotherapy were not included.

Thus, to extend the results of the existing reviews and meta‐analyses mentioned above that were focusing on specific treatment approaches and diagnosis categories [28, 29, 30, 31, 32, 33, 34], a broad and transitional definition of ET is used in this systematic review to truly identify the potential of ET on the reduction of BE in several psychological treatments comprising different therapy approaches. This will help to identify ER as a common underlying mechanism of change in the treatment of BE and provide a comprehensive overview of ET approaches. Moreover, Gross [36, 37] established a process model including four categories of ER, namely, situation selection/modification, attentional deployment, cognitive change, and response modulation. This might be a good starting point to find a clearer and more theory‐driven definition for ET. Therefore, we investigate in the current review, if the included studies match this framework. To further expand evidence, we investigate not only the efficacy of ET on BE (primary outcome) and general ED pathology, but also emotion‐related variables and their interrelationships. We further investigate pre‐assessments and post‐assessments as well as follow‐up (FU) assessments to explore potential maintenance of effects. Contrary to foregoing reviews [28, 29, 30, 31, 32, 33, 34], we investigate transdiagnostic ED samples with regular BE over the whole weight spectrum to investigate the impact of BMI, and we exclude samples without BE, as patients with and without BE can be clearly distinguished [6]. Further, we include loss of control eating (LOC) that is often a precursor of regular BE in childhood or adolescence [3]. We complement our review with a meta‐analytical approach, where we investigate the change of BE from pre‐treatment to post‐treatment within groups as well as potential influencing factors, first of all BMI, ED diagnosis, treatment type, outcome aspects of BE, age, and sex.

## 2 Methods

This systematic review and meta‐analysis is preregistered at PROSPERO (ID: CRD42023361863) and conducted according to the Preferred Reporting Items for Systematic reviews and Meta‐Analyses (PRISMA) statement [38].

### 2.1 Eligibility Criteria

Eligibility criteria are based on the following “PICOS” criteria [39]:

- P (Population): Patients with regular BE episodes or LOC of any age and weight status, that is, patients with BED, BN, anorexia nervosa binge‐purge type (AN‐BP), other specified (OSFED), or unspecified (UFED) EDs as a primary disorder
- I (Intervention): emotion‐related interventions, treatments, and trainings; emotion‐related means psychological treatments that directly target emotions, for example, where core interventions foster emotional awareness or adaptive ER strategies (see examples at Table 1).
- C (Comparison): a control group (active treatment or waitlist) or a within‐subjects comparison before and after treatment
- O (Outcomes): The mandatory primary outcome in the systematic review is BE/LOC in self‐report or as expert rating before and after treatment, for example, frequency, abstinence rate, or rating score. Secondary outcomes pre‐treatment to post‐treatment are (a) general ED psychopathology, for example, from self‐report or expert rating scales; (b) emotion‐related outcomes, for example, assessments of ER, mood status, or assessments of the relationship between emotions and eating; (c) the relationship between emotion‐related outcomes and treatment outcomes; and (d) all respective outcomes at FU.
- S (Study design): clinical studies, for example, randomized controlled trials (RCTs), pre‐post‐comparisons; exclusion of prevention studies, medication studies, ongoing studies, case studies, secondary analyses, and thesis

**TABLE 1:** Examples for emotion‐related treatments for patients with BE.

Approach	Treatment examples	Emotion‐related core elements	Main ER categories^a^ according to Gross' process model [36, 37]	Treatments of included studies
Third wave approaches	Dialectic behavioral therapy (DBT) [101]	Focuses on the acquisition of emotion regulation skills that are intended to perceive mindfully, understand and regulate emotions that trigger BE	Primarily targets *response modulation* strategies (e.g., emotion analysis) combined with *attentional deployment* (e.g., mindfulness), and *situation selection (*e.g.*, diary card)* as well	DBT [50, 59, 60, 64, 73, 77, 78, 80] DBT‐BED [53, 58, 61, 68, 69, 70, 76] Appetite‐focused DBT [47]
	Mindfulness and acceptance‐based approaches [102]	Encourage to open up to the emotional experience by observing emotional states and impulses to BE without acting on them (mindfulness)	Primarily targets *response modulation* strategies (e.g., emotion analysis) combined with *attentional deployment* (e.g., mindfulness), and *situation selection (*e.g.*, diary card)* as well Focus primarily on *attentional deployment and response modulation*	Mindfulness‐based eating awareness training [51] Mindfulness‐based meditation [74] Mindfulness and acceptance based treatment [48] Mindfulness and compassion‐based self‐help intervention [45]
	Self‐compassion approaches [103]	Encourage to open up to the emotional experience by adopting a loving attitude towards oneself instead of shame and guilt and BE (self‐compassion)	Integrate techniques of *attentional deployment*, *cognitive reappraisal*, and *response modulation*	Compassion‐focused self ‐help [49]
	Schema therapy [104]	Addresses ER by overcoming adverse early experiences and its dysfunctional meta‐cognitive schemas and modes that affect BE	Emphasizes *attentional deployment* and *cognitive reappraisal* and targets *response modulation* by working with emotional modes and experiential techniques	Schema therapy [54]
Cognitive behavioral approaches	Integrative cognitive‐affective therapy (ICAT) [35] and other CBT interventions focusing on ER	Emphasize the relationship between emotional states and BE by classical CBT elements, that is, emotion education, identification of emotions associated with BE and adapting strategies.	Place special emphasis on *cognitive reappraisal*, supported by behavioral strategies regarding *situation selection/modification* and *response modulation*	ICAT [55, 62, 72] Emotion‐focused implementation intentions [63] Psychoeducation “Affect School” [56]
Humanistic approach	Emotion‐focused therapy (EFT) [105]	Addresses ER by identifying and accepting primary emotions and underlying needs from secondary emotions, accessing adaptive emotions and transforming maladaptive emotional schemas	Primarily targets *attentional deployment*, while also addressing *response modulation and cognitive reappraisal*	EFT [46, 65, 81]
Treatments combining several approaches	See above/n.a.	See above/n.a.	See above/n.a.	Acceptance‐based behavioral treatment [71] Family‐based therapy and DBT [79] Integrative response therapy [75] Emotional and social mind training [52] Affect regulation training [44] EFGT and dietary counselling [66] ImpulsE (group treatment and inhibitory control training [57] Serious video game and CBT [67]

Abbreviations: CBT = cognitive behavioral therapy; ER = emotion regulation.

^a^ All approaches integrate multiple stages of Gross’ process model of emotion regulation; only the key components are described in the table (displayed in italic letters).

### 2.2 Information Sources and Search Strategy

According to the eligibility criteria, we searched for potentially suitable studies including the topics “binge eating,” “emotion,” and “treatment” at the scientific databases PubMed, PsycInfo, and the Cochrane Central Register of Controlled Trials published up to March 2024 and performed a manual search where we asked experts in the field and checked reference lists. Detailed search terms are available at Supplementary S1.

### 2.3 Study Selection and Data Extraction

**FIGURE 1:**
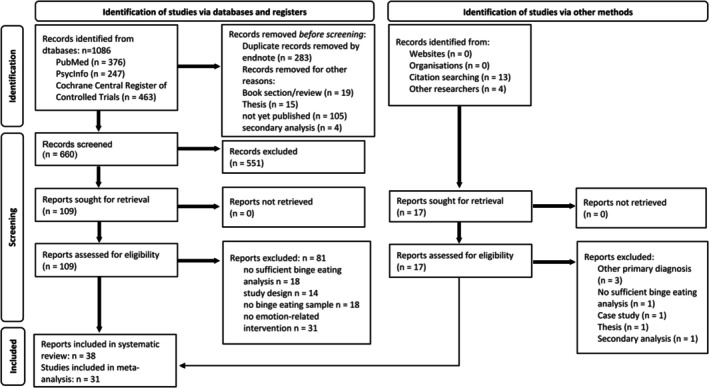
Flow chart of study search according to the PRISMA statement (Page et al., 2021).

Search results from the databases (*N* = 1068) were imported to Endnote, where duplicates, book sections, theses, and ongoing trials were removed (see Figure 1). Remaining articles (*N* = 660) were screened independently according to title and abstract by four authors (JW, MP, KS, TL) regarding potential eligibility. Inconclusive results of the screening, for example, in case of incomplete information or disagreement between authors, were discussed and a decision by consensus was made. Next, full texts of potentially eligible articles (*N* = 109) were independently double‐rated by four authors (AB, FH, LD, TL) regarding inclusion in the review based on the eligibility criteria. Inconclusive results of the eligibility rating were discussed, and a final decision was made by the first author (KS).

For the manual search, HP screened reference lists from systematic reviews and meta‐analyses mentioned above, and experts from the field offered further potentially suitable articles. Two authors (HP, VR) independently rated full texts of those articles (*N* = 17) regarding eligibility and inconclusive studies were rated by the first author (KS).

Data extraction was conducted separately by eight authors (JW, FH, EJL, VR, MP, LD, HP, TL, KS) with an a priori developed data extraction document including the following information: (i) sample characteristics (sample size, diagnosis, subgroups, BMI, age, sex); (ii) characteristics of ET (type, dose, setting, sample size); (iii) control group (type, dose, setting, sample size); (iv) instruments to measure BE/LOC and general ED psychopathology; (v) instruments to measure emotion‐related outcomes; (vi) summary of main findings at the end of treatment: If the study included a control group, comparisons between ET and the control group regarding change of outcomes from pre‐treatment to post‐treatment are reported; if the study included no control group, within‐group changes from pre‐treatment to post‐treatment are reported; (vii) summary of main findings at FU and time points of FU.

Additionally, study quality was independently double‐rated according to EPHPP criteria [40] by all authors. Ratings included the components A Selection bias, B Study design, C Confounders, D Blinding, E Data collection method, F Withdrawals and dropouts, and G Global Rating.

### 2.4 Meta‐Analyses

Several meta‐analyses were conducted by EJL and FB. First, a meta‐analysis for the primary outcome, that is, the change in BE frequency from pre‐treatment to post‐treatment, was conducted. Missing values for this outcome were requested from authors of included studies by KS. Studies reporting no BE frequency were excluded from the meta‐analysis to reduce the heterogeneity of the data. Due to data availability, only the within‐group change of BE frequency pre‐treatment versus post‐treatment could be used for the meta‐analytical approach. Studies were classified as outliers when the estimated effect size's confidence interval did not overlap with the confidence interval of the pooled effect.

Second, meta‐regressions were performed for BMI, age, and sex as potential influencing factors. Further, several subgroup analyses for different ED diagnoses, treatment types, and different aspects of outcome variables assessing BE frequency (i.e., time frame of assessment and instrument) were conducted. We tested for risk of publication bias by Egger's regression test for funnel plot asymmetry [41] and corrected using the trim‐and‐fill method [42].

## 3 Results

The PRISMA flow diagram of study inclusion is presented at Figure 1. Finally, 38 articles were included in the systematic review. Interrater reliability of eligibility ratings was substantial with a weighted Cohen's kappa of *κ* = .67 [43].

**FIGURE 2:**
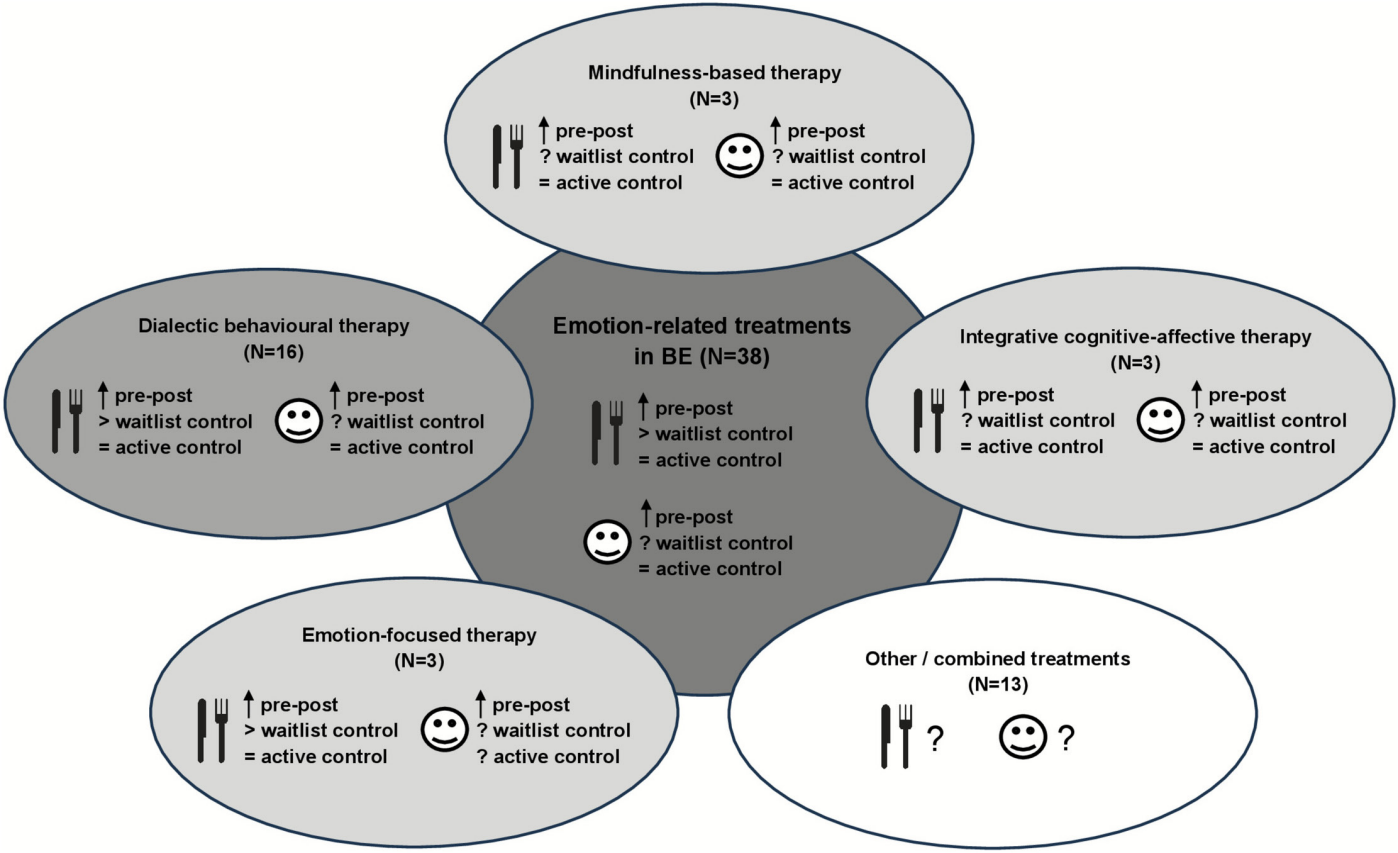
Visual summary of emotion‐related treatments. Described are emotion‐related treatments separated by treatment sub‐categories, and its evidence regarding changes in BE frequency (fork and knife pictogram) as well as emotion‐related outcomes (smiley pictopgram) at end of treatment. The strength of the greytone is indicating the level of evidence regarding pre‐post‐comparisons and comparisonwith waitlist and active control groups.

A detailed description of each included study and summary of the results is presented at Supplementary S2. Of the 38 included studies, 20 were RCTs [44, 45, 46, 47, 48, 49, 50, 51, 52, 53, 54, 55, 56, 57, 58, 59, 60, 61, 62, 63]. Twenty‐six studies compared ET with a control group, that is, *N* = 16 to active treatment(s) [48, 50, 52, 54, 55, 56, 57, 58, 62, 63, 64, 65, 66, 67, 68, 69], *N* = 7 to a waitlist control [44, 45, 46, 47, 53, 59, 61], *N* = 3 to both [49, 51, 60], and *N* = 12 had a within‐group design [70, 71, 72, 73, 74, 75, 76, 77, 78, 79, 80, 81]. Thirty‐one of the 38 studies delivered sufficient data to be included in the meta‐analysis regarding BE frequency. The sample size varied strongly between 10 and 189 participants with a high proportion of women (usually 80%–100%). Regarding weight status, 25 studies included overweight and obese samples, six studies included normal weight samples, and in seven studies, BMI was not reported. Most frequently, adult patients with BED were examined (*N* = 18), followed by mixed samples with different diagnoses (*N* = 14) and patients with BN (*N* = 6). Only five studies examined adolescents. The most frequently investigated ET approach was DBT (*N* = 16), followed by CBT focusing on emotions (*N* = 5) [55, 56, 62, 63, 72], and several studies used combined treatment approaches (*N* = 8) [44, 52, 57, 66, 67, 71, 75, 79]. More details regarding the type of treatment are presented at Table 1 and Figure 2. Overall, evidence regarding changes in BE and emotion‐related outcomes pointed in the same direction independent of the treatment type (see Figure 2). Regarding the categories provided by the process model of Gross [36, 37], it became evident that most of the identified treatments address several of the postulated categories from Gross, but with different focuses or in different ways (see Table 1).

### 3.1 Ratings of Study Quality

Table 2 gives an overview of the EPHPP ratings. Interrater reliability was excellent with a weighed Cohen's kappa of *κ* = 0.94. The study quality was rated as weak (“3”) in 18 studies, as moderate (“2”) in nine studies, and as strong (“1”) in 11 studies (*M* = 2.2). Notably, four of the five studies evaluating adolescent populations were assessed to be of weak methodological quality [77, 78, 79, 80]. In general, the weakest ratings were achieved in the category “D blinding” (*M* = 2.6), which means that in most studies, assessors have been aware of the treatment status of the patients. Another high proportion of studies had weak or moderate ratings at the category “F Withdrawals and Dropouts” (*M* = 2.0) indicating a high treatment attrition or incomplete report of drop‐outs. In contrast, most studies achieved strong ratings in the category “E Data Collection Method” (*M* = 1.1) and several studies showed a strong study quality, for example, several RCTs [52, 53, 55, 57, 62, 82].

**TABLE 2:** Study quality of included studies according to EPHPP criteria.

	Rating 1							Rating 2							Final decision both raters
Study	A	B	C	D	E	F	G	A	B	C	D	E	F	G	
Berking et al. (2022)	2	1	1	2	1	2	1	2	1	1	2	1	1	1	1
Blood et al. (2020)	2	2	3	2	1	2	2	1	2	3	2	1	2	2	2
Chen et al. (2017)	2	1	1	2	1	1	1	1	1	1	2	1	2	1	1
Compare et al. (2016)	2	2	2	3	1	3	3	2	1	1	3	2	3	3	3
Compare et al. (2013)	3	2	1	3	1	1	3	3	2	2	3	1	1	3	3
Duarte et al. (2017)	2	1	2	3	1	1	2	1	1	2	3	1	2	2	2
Fernandez‐Aranda et al. (2015)	3	2	1	3	1	3	3	3	2	2	3	1	2	3	3
Fischer & Peterson (2015)	2	2	3	3	1	2	3	3	2	3	3	1	2	3	3
Glisenti et al. (2021)	1	1	1	2	1	1	1	1	1	1	2	1	1	1	1
Hill et al. (2011)	2	1	1	1	1	2	1	1	1	1	1	1	2	1	1
Juarasico et al. (2017)	1	2	3	3	1	3	3	1	2	3	3	1	3	3	3
Juarasico et al. (2021)	1	1	1	3	1	3	3	1	1	1	3	1	3	3	3
Juarasico et al. (2020)	3	2	3	3	1	3	3	2	2	3	3	1	3	3	3
Kamody et al. (2019)	3	3	3	3	1	3	3	3	3	3	3	1	3	3	3
Kelly & Carter (2015)	2	1	1	3	1	2	2	2	1	1	3	1	2	2	2
Klein et al. (2012)	3	2	3	3	1	3	3	3	2	3	3	1	3	3	3
Klein et al. (2013)	3	1	3	3	1	3	3	3	1	3	3	1	3	3	3
Kristeller et al. (2014)	3	1	3	3	1	2	3	2	1	3	3	1	2	3	3
Kristeller & Hallett (1999)	3	2	2	3	2	2	3	3	2	2	3	2	2	3	3
Lammers et al. (2020)	2	2	2	2	2	2	1	2	1	2	1	1	2	1	1
Lammers et al. (2022)	2	2	2	3	2	2	2	2	2	2	3	1	2	2	2
Lavender et al. (2012)	1	1	1	2	2	2	1	2	1	1	1	1	2	1	1
Masson et al. (2013)	2	1	1	2	2	2	1	2	2	1	2	1	2	1	1
McIntosh et al. (2016)	1	1	1	3	1	2	2	2	1	1	3	1	2	2	2
Murray et al. (2015)	3	2	3	3	1	1	3	2	2	1	3	1	3	3	3
Peterson et al. (2020)	2	1	1	2	1	1	1	1	1	1	2	1	2	1	1
Petersson et al. (2022)	3	1	3	3	1	1	3	3	1	3	3	1	1	3	3
Preuss et al. (2017)	1	1	2	1	1	2	1	1	1	2	1	1	2	1	1
Robinson et al. (2013)	3	2	1	3	1	2	3	2	2	3	3	1	2	3	3
Safer et al. (2001)	2	1	2	3	1	1	2	2	1	2	3	1	1	2	2
Safer et al. (2010)	1	1	1	3	1	1	2	1	1	1	3	1	1	2	2
Salbach et al. (2007)	2	2	3	3	1	1	3	2	2	3	3	1	1	3	3
Salbach‐Andrae et al. (2009)	2	1	1	2	1	2	1	2	1	1	2	1	1	1	1
Tanis et al. (2023)	2	1	2	3	1	2	2	2	1	2	3	1	2	2	2
Telch et al. (2000)	3	2	3	3	1	1	3	3	2	3	3	1	1	3	3
Telch et al. (2001)	3	1	1	2	1	2	2	3	1	2	2	1	3	3	2
Wnuk et al. (2015)	2	3	3	3	1	1	3	2	3	3	3	1	2	2	3
Wonderlich et al. (2014)	2	1	1	2	1	1	1	3	1	1	2	1	1	2	1
Total mean	1,9	1,5	1,7	2,6	1,2	2,0	2,2	1,9	1,5	1,9	2,6	1,0	2,0	2,1	2,2

*Note:* A, selection bias; B, study design; C, confounders; D, blinding; E, data collection method; F, withdrawals and dropouts; G, global rating, 1 = strong, 2 = moderate, 3 = weak.

### 3.2 BE (Primary Outcome)

#### 3.2.1 Synthesis of Qualitative Results Regarding BE

As can be seen in Table S2, BE was assessed in most cases (*N* = 19) by Eating Disorder Examination (EDE) [83] representing the current gold standard of ED assessment. Others used the Eating Disorder Inventory (EDI) [84] or the Structured Interview for Anorexia Nervosa and Bulimia Nervosa (SIAB) [85] as interviews, or self‐reports, most often the EDE‐Q [86] (*N* = 25). Most of the studies assessed the frequency of BE (*N* = 27), followed by BE days (*N* = 6) [49, 51, 57, 58, 64, 75]. Two studies assessed LOC including subjective BE [48, 72], two assessed abstinence rates [65, 67], and one assessed the portion size of BE [80].

Table 3 gives an overview of the qualitative synthesis of the results. In all studies investigating the change of BE within‐groups (*n* = 12), BE was reduced from pre‐treatment to post‐treatment [70, 71, 72, 73, 74, 75, 76, 77, 78, 79, 80, 81]. Regarding studies with a waitlist control, seven studies compared ET with a waitlist [44, 45, 46, 47, 53, 59, 61] and three to an active and a waitlist control [49, 51, 60]. In all of these 10 comparisons, BE was reduced more strongly in the ET compared with waitlist. Regarding the studies with an active control group, 12 studies used one active control group [48, 50, 52, 55, 56, 57, 58, 62, 66, 67, 68, 69], four studies used two active control groups [54, 63, 64, 65], and three studies used one active and one waitlist control [49, 51, 60], resulting in 23 comparisons of ET with an active control. Out of those, 16 showed comparable effects of ET with an active control regarding BE reduction [48, 49, 51, 52, 54, 55, 57, 60, 62, 63, 64, 65, 66, 67, 69], four comparisons showed inferiority of ET [56, 64, 65, 68], and three superiority [50, 58, 63]. In the three superior studies, rather weak control groups were used, and in the four inferior studies, rather strong control groups were used (see Table S2). To summarize, the qualitative results regarding the efficacy of ET towards BE indicate that ET is able to reduce BE, favorable to no treatment and comparable with other treatments.

**TABLE 3:** Overview of study results from the qualitative review regarding BE and emotion‐related outcomes.

	Study design	EOT	FU
BE	Pre vs. post:	12 improvement	4 further improvement 4 maintenance 2 deterioration
	WC:	10 ET >	3 ET >
	AC:	16 ET =	19 ET =
		3 ET >	9 ET >
		4 ET <	1 ET <
Emotion regulation	Pre vs. post:	5 improvement 1 mixed results	1 further improvement
	WC:	3 ET >	1 ET >
		2 ET =	1 further improvement
		1 mixed results	1 mixed results
		1 no change to baseline	
	AC:	7 ET =	6 ET =
		1 ET <	2 ET <
		1 no change to baseline	1 ET >
Relation of emotions and eating	Pre vs. post:	4 improvement	2 further improvement
		1 mixed	1 no change
		1 no change	
	WC:	2 ET =	
		1 mixed	
	AC:	1 ET =	2 ET =
		1 ET <	1 ET <
		1 ET >	

Abbreviations: AC, comparison between ET and the active control group; EOT, end of treatment; ET, emotion‐related treatment; FU, follow‐up; pre vs. post, within group changes from pre‐treatment to post‐treatment in studies without a control group; WC, comparison between ET and the waitlist control group.

#### 3.2.2 Meta‐Analyses and Potential Influencing Factors

**FIGURE 3:**
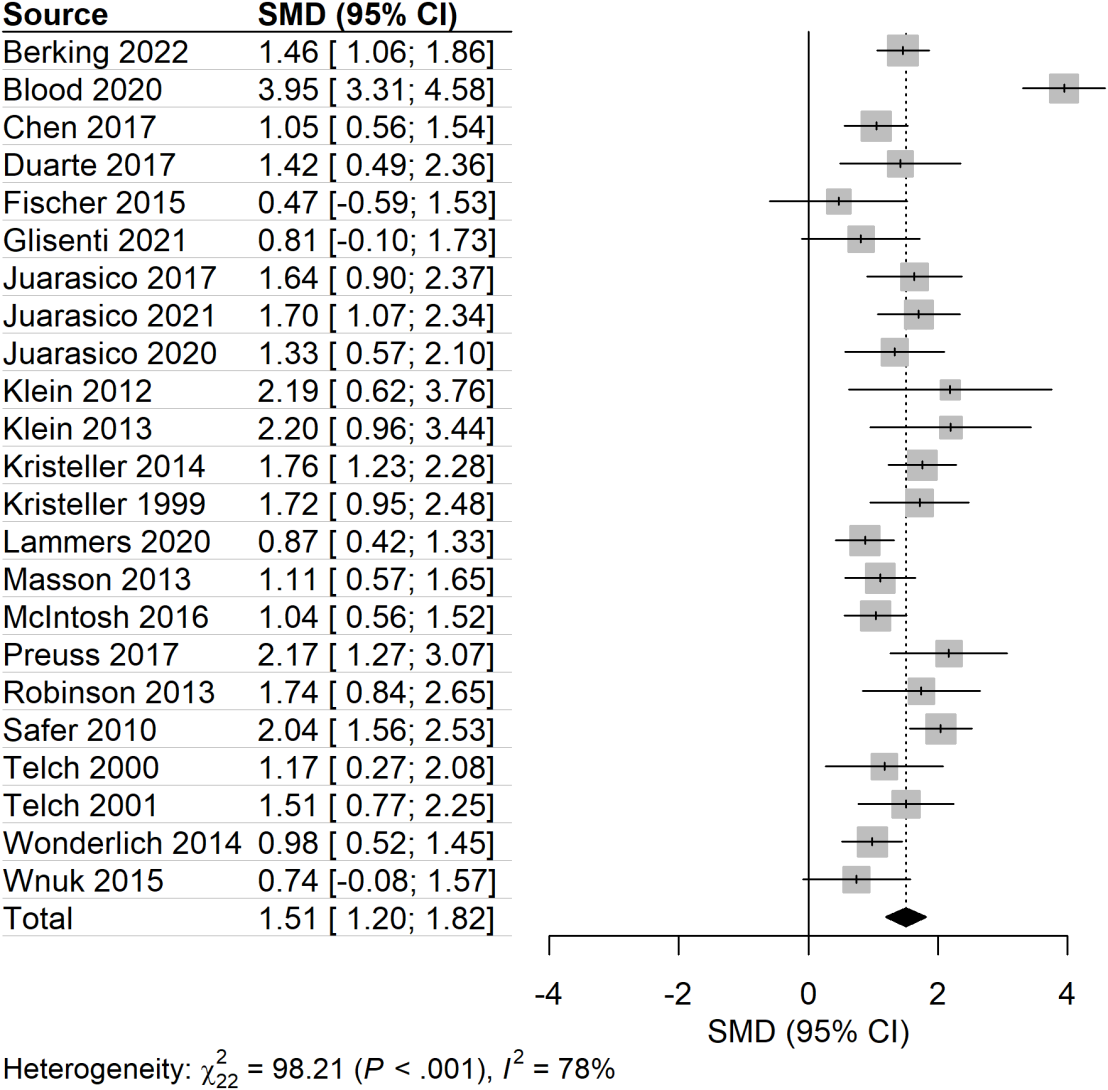
Within‐group changes of BE frequency from pre to posttreatment. CI, confidence interval; SMD, standardized mean difference.

Figure 3 gives an overview of the results in the meta‐analysis regarding within‐group changes of BE frequency pre‐ET versus post‐ET. Out of the *N* = 31 studies that were included in the meta‐analysis, *N* = 8 were excluded because they were classified as outliers. After outlier removal, *standardized mean difference (SMD)* and study‐heterogeneity decreased, while still remaining significant. In line with the qualitative synthesis, the results presented here indicate a strong reduction of BE from pre‐treatment to post‐treatment within groups. Egger's regression test did not indicate significant funnel plot asymmetry (*p* = 0.51). Consequently, explorative trim and fill corrections did not change the observed effect size.

The analyses of potential influencing factors by meta‐regressions revealed that there was no impact of BMI (*M* = 33.34 kg/m^2^, *SD* = 5.09; *b* = 0.10, *p* = 0.058), age (*M* = 38.01, *SD* = 8.29; *b* = −0.01, *p* = 0.60), and sex (*M* = 95.83% females, *SD* = 6.71; *b* = −0.003, *p* = 0.74) on BE reduction in ET. Further, different types of diagnoses did not affect the amount of BE reduction (*p* = 0.52; see Supplementary S3a). In particular, studies with BED samples against studies with mixed samples or other diagnoses did not show a significant difference (*p* = 0.25; see Supplementary S3b). Additionally, different types of ET did not influence the amount of BE reduction (*p* = 0.67; see Supplementary S3 c)). In particular, testing DBT versus other treatments did not show a significant difference (*p* = 0.53; see Supplementary S3d). Taking into account the different outcome variables used to assess BE frequency, we did not find any significant difference between different time frames (28 days vs. 7 days vs. not reported, *p* = 0.56; see Supplementary S3e) and instruments (EDE vs. other vs. not reported, *p* = 0.19; see Supplementary S3f). Thus, none of the potential influencing factors showed a significant impact on BE reduction in ET. We did not detect obvious differences depending on those variables in the qualitative analysis as well (see Table S2).

#### 3.2.3 Maintenance of Effects

Twenty‐five studies had FU assessments and used heterogeneous, and sometimes several time spans from 1 to 12 months (see Table S2). In the 10 available pre‐post‐comparisons, the reduction of BE was maintained at FU in four comparisons [70, 75, 76], further improved in four comparisons [53, 71, 77, 78], and worsened in two comparisons [61] (see Table 3). ET was superior to a waitlist control at FU in all three FU comparisons [44, 45, 51]. Out of the 29 comparisons of ET with an active control at FU within 15 studies, BE reduction was comparable in ET at the FU in 19 comparisons [48, 52, 54, 55, 57, 58, 62, 63, 64, 66, 68, 69], superior in nine comparisons [51, 56, 63, 64, 65] and inferior in one comparison [65]. As in the post‐treatment results, rather weak control groups were used in the superior comparisons (besides [64]), and in the inferior comparison, a rather strong control group was used. Taken together, results were overall maintained at FU up to 12 months [54, 56, 58, 64], favorable to no treatment and comparable with an active control.

### 3.3 General ED Psychopathology

According to Table S2, general ED psychopathology was assessed in most cases (*N* = 25) by EDE or EDE‐Q [86], but other instruments like Binge Eating Scale (BES, *N* = 7) [87] or Eating Disorder Inventory (EDI, *N* = 5) [84] were used several times as well. Compensatory behaviors were reported in studies investigating patients with bulimic symptoms [47, 52, 54, 59, 60, 62, 77, 79, 80, 81]. According to Table S2, results regarding general ED psychopathology and compensatory behaviors highly overlapped with the results regarding BE. Regarding general ED psychopathology, differences occurred only in six studies post‐treatment [49, 51, 56, 63, 68, 69] and at FU in four studies [51, 58, 63, 68], and regarding purging, only one study delivered different results from BE [81]. Overall, the results build a consistent picture indicating that ET is reducing ED psychopathology, favorable to no treatment and comparable with other treatments.

### 3.4 Emotion‐Related Outcomes

According to Table S2, emotion‐related outcomes were assessed by self‐reports in 26 studies in very heterogeneous ways. An overview of the used ER instruments is given at the supporting information S4 in a table. Most of the studies (*N* = 22) addressed ER, for example, by the Difficulties in Emotion Regulation Scale (DERS) [88], Negative Mood Regulation Scale (NMRS) [89], or they assessed specific ER strategies like mindfulness, acceptance, or self‐compassion [45, 48, 49, 71]. Regarding these ER studies, five pre‐post‐comparisons within the ET group showed improvements at the end of treatment [72, 76, 77, 79, 81], and one showed mixed results [71] (see Table 3). In comparison with a waitlist control, three studies reported superiority [44, 49, 53], two studies comparability [47, 59], one study mixed results [45], and one study no changes compared with baseline [61]. In comparison with an active control group, seven studies reported comparable results of ET [48, 52, 55, 56, 57, 62, 68], one inferiority [69], and one no changes compared with baseline [58]. At FU, the only study that examined ER from pre‐ to postreatment reported further improvement [77]. The three studies with a waitlist control reported further improvement [53], superiority of ET [44], or mixed results [45] at FU. In comparison with an active control group at FU, six studies reported comparability with an active control group [48, 52, 55, 57, 62, 68], one superiority [56], and two inferiority [58, 69]. Overall, these studies indicate that ER can be improved by ET and that ET is comparable in effectivity to active treatments. However, compared with BE and general ED psychopathology as outcomes, fewer studies investigated ER and the results are somewhat more inconsistent, especially in comparisons of ET with a waitlist control. It might be that these inconsistencies derive from different self‐report instruments assessing ER. For instance, in studies using the DERS, there was a tendency for results favoring ET [48, 53, 55, 56, 62, 72, 79] versus negative results [58, 71]. In studies using the NMRS, there was a tendency for negative [47, 58, 59, 61] versus positive [76] results.

Further, several studies (*N* = 12) assessed (additionally to ER) the relationship between emotions and eating, most of them emotional eating, for example, with the Emotional Eating Scale (EES) [90] or the Dutch Eating Behavior Questionnaire (DEBQ) [91], but mindful eating was assessed as well [71, 74]. Regarding this, four out of six studies reported improvements in pre‐post‐comparisons [70, 71, 74, 75, 76, 78], but results were mixed in comparisons with a waitlist control [47, 59, 61], and with an active control [58, 68, 69] (see Table 3). At FU, results were scarce and mixed (pre–post [70, 75, 78], active control [58, 68, 69]) as well. Results did not differ depending on assessments, and due to this few and inconsistent evidence, we were not able to draw conclusions regarding the efficacy of ET on the relationship between emotions and eating. In addition, current mood status [47, 58, 59, 61, 76] and diverse other emotion‐related outcomes [52, 56, 81] were assessed. The evidence regarding these outcomes is scarce and inconsistent (see Table S2), so we did not draw any conclusions.

### 3.5 Relationship Between Emotion‐Related Outcomes and Treatment Outcome

Some of the studies included in this review (*N* = 6) examined potential mechanisms of change, that is, the relationship between emotion‐related outcomes and treatment outcome (see Table S2). Of these, only one study conducted a mediation analysis [44], two studies tested a moderation effect [49, 52], and three studies explored correlation analyses [51, 71, 74]. With regard to the results, Berking and colleagues [44] report that improvement in ER skills served as a mediator of BE reduction and general ED psychopathology. Juarascio and colleagues [71] detected strong associations between improvement in acceptance and ER strategies with improvement in general ED psychopathology. Kelly and Carter [49] report that patients with low fear of self‐compassion benefited more from a compassion‐focused self‐help approach in terms of general ED psychopathology. Kristeller and colleagues [51, 74] demonstrated that the amount of mindfulness training was associated with the improvement in BE pathology. Further, improvement in the sense of eating control, sense of mindfulness, and awareness of satiety cues was related to BE reduction [74]. However, beliefs about emotions and distress tolerance represented no moderators of change toward general ED psychopathology [52]. Taken together, these analyses imply that improvements in ER and mindfulness might represent important mechanisms of change in BE treatment. However, the shortage of studies that statistically or formally tested the mediating or moderating role of changes in ER on BE outcomes limits the strength of this interpretation.

## 4 Discussion

In this systematic review and meta‐analyses, we investigated the efficacy of ET in patients with BE regarding BE, general ED psychopathology, and emotion‐related outcomes across the life span. Further, we investigated the influence of BMI, ED diagnosis, treatment type, outcome aspects of BE, age, and sex on change in BE. Overall, our results suggest that ET is promising in treating BE. They show significant symptom reductions after treatment, superiority compared with waitlist control groups, and comparability with active control groups in terms of improvements in BE, general ED psychopathology, and ER. The effects appear to be stable at FU analyses for 1 up to 12 months. None of the investigated potential factors affected the efficacy of ET.

Regarding BE and general ED psychopathology, our qualitative synthesis shows consistent evidence that ET are able to reduce symptomatology from pre‐treatment to post‐treatment, are favorable to no treatment, and comparable with other active treatments. For instance, ET seem to be superior to waitlist controls and low‐level interventions [50, 58] and comparable with CBT, whereas ET add‐ons to TAU did not seem to improve treatment outcomes remarkably [56, 67]. Moreover, the results from the meta‐analysis indicate in line with the qualitative analyses that BE frequency reduces strongly from pre‐treatment to post‐treatment, even after exclusion of outliers. However, as we were only able to compute pre‐post‐comparisons within groups, the meta‐analytic results have to be interpreted with caution. When FU assessments were reported, patients were able to maintain their progress after ET up to 12 months [54, 56, 58, 64]. Thus, we conclude from the current results that ET seem to be an effective approach to treat patients with BE.

Regarding potential mechanisms of change, we derived from several ER models [16, 19, 20] that ET might improve ER in patients with BE and that this might lead to reduced BE. In line with this hypothesis, the investigated studies indicate that ET are able to improve ER from pre‐treatment to post‐treatment and are comparable with other active treatments, even at FU. Thus, ET might be useful to improve ER. Furthermore, several studies in this review [44, 49, 51, 71, 74] identified associations between improvements in ER and reductions of BE or general ED psychopathology. This indicates that especially improvement in ER strategies, acceptance of emotions, and mindfulness might represent potential mechanisms of change in ET regarding BE treatment. However, without rigorous mediation or moderation analyses, conclusions about ER improvements as a mechanism underlying reductions in BE remain tentative. Future research should employ RCTs or experimental studies specifically designed to test this hypothesized link, using appropriate statistical models to clarify the causal role of ER in BE treatment outcomes. Additionally, we would have expected that ET, that is, treatments that are conceptually designed to have a high impact on emotions, might even show stronger influence on emotion‐related outcomes compared with other active treatments like classical CBT though both treatments produced comparable results [48, 55, 62, 68]. One reasonable explanation might be that in classical CBT, emotions are influenced as well, though in a more indirect way via modification of behavior and cognitions. It should also be noted that treatment adherence was not assessed in most of the studies, which could ensure to what extent and how often ER actually played a role in the therapy, and whether the active control groups also implemented ER interventions or not. Another important point is to keep in mind that fewer studies (*N* = 22) investigated ER compared with studies investigating BE pathology (*N* = 38), and especially in studies with waitlist controls, evidence was somewhat inconsistent. As various self‐report instruments have been used to assess ER, we speculate that these heterogeneous methods might have contributed to the more inconsistent results. For example, DERS [88] investigates various aspects of emotion dysregulation like emotional awareness, ER strategies, acceptance, and impulse control, whereas NMRS [89] investigates specifically expectations regarding the ability to regulate negative emotions. Thus, as ET addresses ER in a broader context and does not only focus on ER strategies (see Table 1), a broader assessment of ER such as the DERS might be more useful to identify emotion‐related changes by ET. Moreover, foregoing studies speak for a high reliability and validity of the DERS [88], some of the other used instruments have already been criticized regarding their reliability and validity [92], some studies in this review used unvalidated instruments [74, 81], and 12 studies did not assess ER in any way (see Table S2). Therefore, it might be useful to use DERS as a rather global measure in addition to more specific instruments. Further, 12 studies investigated the relationship between emotions and eating behavior and delivered inconsistent results. It might be that the relationship between emotions and eating behavior is better investigated by a manipulation of the emotion (e.g., mood induction) and subsequent assessments of eating behavior. Another option would be to assess the use of ER strategies and related eating behavior in daily life, for example, by ecological momentary assessments (EMA) [93]. Overall, future intervention studies should investigate suggested mechanisms of action more thoroughly.

Regarding potential influencing factors of the results presented here, BMI, ED diagnosis, type of ET, outcome aspects of BE, age, and sex do not seem to influence the efficacy of ET. However, studies showed high heterogeneity, especially regarding ED diagnosis, treatment types, and outcome aspects, which might induce some bias in the results. To investigate such potential biases, we conducted several subgroup analyses and did not find statistical differences. For instance, we found no statistical difference between patients with BED, BN, and mixed samples. This result is opening the discussion for a transdiagnostic treatment of BE. In line, several researchers presented evidence that patients with regular BE episodes represent one entity, independent from the diagnosis, that can be statistically distinguished from patients with restrictive eating behavior [5, 6, 94]. Further, we detected no difference in the meta‐analyses regarding the reduction of BE depending on treatment type, especially between DBT and other ET, though DBT delivered most evidence (see also Figure 2 regarding the qualitative results). This points to the idea that it might be reliable to subsume the investigated treatments under the umbrella term ET. Further, the variability of BE outcome measures used across studies did not influence the reported treatment outcome. One reason for this might be that the different outcome measures bear the potential to be highly correlated. Nevertheless, it would be favorable for further studies to align outcome measures to increase comparability and facilitate interpretation. Taken together, due to the heterogeneity of the data, the presented results have to be interpreted with caution. On top of this heterogeneity, the evidence here regarding potential influencing factors is thin. For instance, several studies reported no data for the analyses of the potential influencing factors and the variance of some comparators (e.g., male subjects, adolescents) was low. In particular, most of the studies investigated overweight and obese samples with a mean BMI of 33, so that ceiling effects might have appeared regarding the potential influence of BMI on treatment outcome. Moreover, nearly no conclusions can be drawn about ET in men or minors with BE. Regarding age, both the meta‐analysis and qualitative analysis suggest no significant differences between minors and adults. However, it is important to note that only five studies investigated ET in adolescents, with just one RCT (see Table S2b)) and generally weak study quality (see Table 3). The limited evidence available indicates that the outcomes in adolescents are consistent with those found in adults. Given that the studies involving adolescents in this review report a mean age of 16 years, it is possible that the most critical developmental changes have already occurred, leading to similar results across age groups. The lack of developmental diversity in the current literature restricts the generalizability of these findings to child and prepubertal populations. This highlights the need for further research, which aligns with the conclusions of a recent scoping review [95]. Overall, more systematic, standardized, and well‐designed studies are necessary to investigate these and other potential influencing factors.

Taken together, this systematic review is the first attempt to investigate a range of ET across ED diagnoses and in adolescents as well as adults. The results are overall promising as they indicate the efficacy of ET toward BE pathology and suggest ER as a potential transdiagnostic mechanism of change. Finally, it might help to include ET treatments in official treatment guidelines. However, future studies should reveal exactly which components of ET are responsible for the positive effects on ED symptomatology.

Regarding limitations, due to paucity or heterogeneous data, it was not possible to compute meta‐analyses regarding the treatment progress in ET compared with that in the control groups, but only regarding within‐group pre to post differences. Though pre‐changes to post‐changes within groups are often reported in meta‐analyses, this limits the conclusions that can be drawn from the meta‐analysis regarding comparative efficacy, as it might lead to bias and overgeneralization [96]. Further, due to data paucity and heterogeneity, no meta‐analyses regarding emotion‐related outcomes or FU data could be computed. Thus, the results of the meta‐analysis should be interpreted with caution and understood as an add‐on to the qualitative synthesis. Indeed, qualitative results of a comparable BE reduction in ET compared with the active control groups and changes in ER complement these results. Further, BE is known to be rather stable if not treated [97, 98], which corroborates the assumption of a treatment‐related reduction as well.

In addition, study heterogeneity was high calling for standardization, for example, standardized measures for BE, but especially for emotion‐related outcomes. Moreover, the results of several studies have to be interpreted with caution due to weak study quality, for example, regarding studies in adolescents. It should be noted that EPHPP criteria show a high degree of standardization, but some of the criteria are very strict and hard to achieve in clinical trials.

Another important limitation of this review is that there exists no conclusive definition of ET, so we had to use an explorative and broad definition of ET including all treatments that directly target emotions. While preparing this review, we realized that the subdivision of ET is complex, because several studies combined treatment approaches [44, 45, 66, 71, 75, 79], and furthermore, some ET conceptually include elements of other ET; for example, DBT includes mindfulness and CBT elements, and schema therapy includes EFT and psychoanalytical elements [27]. Thus, it seems reasonable to summarize treatments focusing on emotions under the umbrella term ET. Others [25, 99] have summarized such treatments already. The summary of these treatments within the term ET is also in line with our meta‐analytic results where we did not find differences between specific ET treatments regarding BE reduction. Moreover, several studies investigated ER and delivered evidence that ER is influenced by ET. Nevertheless, it could be useful to subdivide this broad definition of ET regarding its specific mechanisms of change, for example, by the process model of ER from Gross [36, 37]. Regarding this categorization, we figured out that all identified treatments address several of the postulated categories from Gross. However, they put different focuses on these categories or address them in different ways (see Table 1). For example, it seems as if third wave and humanistic approaches rather focus on attentional deployment and response modulation, for example, by mindfulness exercises, skills training, chair work, or emotion analyses. Further, it seems as if they focus more on a modification of the emotion and less on a cognitive change. Emotion‐related CBT is using more antecedent strategies like situation selection/modification and cognitive reappraisal. This is in line with Linardon et al. [28] who state that third‐wave interventions use response‐focused ER strategies, whereas in CBT, more antecedent‐focused strategies are used that prevent the activation of emotions. Contrary, third wave and humanistic approaches might use more methods that elicit emotions [27, 28]. Overall, this is the first attempt to categorize specific treatments according to Gross' process model. To summarize, ET seems to address several proposed emotion‐related mechanisms with several treatment components. It might be that each of these ET components may demonstrate a unique mechanism of action [25]. Taken together, our working definition of ET is preliminary but seems useful to examine the efficacy of such treatments and their underlying mechanisms of change. It might help to overcome historically evolved boundaries and to find a common ground for treating BE or other mental disorders.

As an outlook, the results of this review have to be further investigated in terms of the proposed definition of ET, the efficacy of ET regarding BE pathology, and potential influencing factors. Though we have not detected specific influencing factors, it might be that specific subgroups respond more to ET and some respond more to other treatments. Moreover, the mechanisms of change underlying ET have to be further investigated and disentangled [25]. Thus, one way forward could be to investigate single proposed mechanisms of change instead of whole treatment concepts. Though ER strategies seem to be one promising approach, emotional awareness is less investigated, but might be an important mechanism of change as well. Furthermore, emotional awareness and ER strategies are sometimes summarized under the term ER and sometimes treated as independent aspects and investigated separately [88]. For this purpose, reliable definitions of emotion‐related concepts have to be elaborated and the used instruments have to be standardized. Hopefully, ongoing trials, for example, [63, 100], will elucidate these open questions.

## 5 Conclusions

Overall, we conclude from this qualitative review and meta‐analysis that ET might be a useful generic term and that ET have been shown to be effective in the treatment of transdiagnostic patients with regular BE. Though more studies are necessary to draw final conclusions, ET are able to reduce BE, general ED psychopathology, and improve emotion‐related outcomes. Especially ER seems to be a promising mechanism of change in ET that is improved after ET and associated with BE pathology. So far, we could not detect strong influences from sociodemographic factors, ED diagnosis, BMI, and treatment type on ET efficacy. Therefore, a further investigation of potential influencing factors and mechanisms of change is necessary.

## Author Contribution

Conceptualization: all authors; data curation: KS; formal analysis: EJL, FB, KS; funding acquisition: KS, TL; investigation: all authors; methodology: all authors; project administration: KS; supervision: TL; validation: all authors; visualization: KS, EJL, FB; roles/writing – original draft: KS; writing – review and editing: all authors.

## Funding

This work was funded by the German Society of Eating Disorders (DGESS e.V.). KS was supported by the Ministry of Science, Research and the Arts Baden‐Württemberg. EJL and FB were supported by the Deutsche Forschungsgemeinschaft (DFG 523716110). We acknowledge support by Open Access Publishing Fund of University of Tübingen.

## Conflicts of Interest

The authors declare no conflicts of interest.

## Supporting information


**Table S1:** Search terms for the literature search in each database.
**Table S2:** (a) Studies in adults.
**Table S2:** (b) Studies in adolescents.
**Table S3:** (a) Subgroup analysis of different diagnoses.
**Table S3:** (b) Dichotomous subgroup analysis of different diagnoses (BED vs. all other diagnoses).
**Table S3:** (c) Subgroup‐analysis of different treatments.
**Table S3:** (d) Dichotomous subgroup analysis of different treatments (DBT vs. all other treatments).
**Table S3:** (e) Subgroup analysis of different time frames in outcome variables.
**Table S3:** (f) Subgroup analysis of different instruments used in outcome variables.
**Table S4:** Overview on used emotion regulation instruments in the identified studies.

## Data Availability

The data that support the findings of this study are available from the corresponding author upon reasonable request.
