# Supplementary material for: Emotion‐Related Treatments in Patients With Binge Eating Episodes—a Systematic Review and Meta‐Analysis
Source: Obes Rev. 2025 Dec 12;27(5):e70058. doi: 10.1111/obr.70058 (PMC13070887; doi:10.1111/obr.70058)
Supplement: Supplementary file 1 — Table S1: Search terms for the literature search in each database. Table S2: (a) Studies in adults. Table S2: (b) Studies in adolescents. Table S3: (a) Subgroup analysis of different diagnoses. Table S3: (b) Dichotomous subgroup analysis of different diagnoses (BED vs. all other diagnoses). Table S3: (c) Subgroup‐analysis of different treatments. Table S3: (d) Dichotomous subgroup analysis of different treatments (DBT vs. all other treatments). Table S3: (e) Subgroup analysis of different time frames in outcome variables. Table S3: (f) Subgroup analysis of different instruments used in outcome variables. Table S4: Overview on used emotion regulation instruments in the identified studies. [file OBR-27-e70058-s001.pdf]

Supporting information to

EMOTION-RELATED TREATMENTS IN PATIENTS WITH BINGE EATING EPISODES – A SYSTEMATIC REVIEW AND META-ANALYSIS

Kathrin Schag [a, b*], Jessica Werthmann [c], Elisabeth Johanna Leehr [d], Hanna Preuss-van-Viersen [e], Eva-Maria Skoda [f,g], Vanessa Rentrop [f,g], Fabian Breuer [d], Maarit Pelzer [c], Laura Derks [h], Florian Hammerle[e], Arne Bürger [i, j], Tanja Legenbauer [h]

[a] Department of Psychosomatic Medicine and Psychotherapy, University Hospital Tübingen, Tübingen, Germany

[b] Centre of Excellence for Eating Disorders Tübingen (KOMET), Tübingen, Germany

[c] Albert-Ludwigs University of Freiburg, Institute of Psychology, Clinical Psychology and Psychotherapy Unit, Freiburg, Germany

[d] Institute for Translational Psychiatry, University of Münster, Münster, Germany

[e] Department of Child and Adolescent Psychiatry and Psychotherapy, University Medical Center of the Johannes Gutenberg University Mainz, Mainz, Germany

[f] Clinic for Psychosomatic Medicine and Psychotherapy, LVR-University Hospital Essen, University of Duisburg-Essen, Essen, Germany

[g] Center for Translational Neuro- and Behavioural Sciences (C-TNBS), University of Duisburg-Essen, Essen, Germany

[h] LWL-University Hospital for Child and Adolescent Psychiatry, Psychotherapy and Psychosomatics, Ruhr University Bochum, Hamm, Germany

[i] Department of Child and Adolescent Psychiatry, Psychosomatics and Psychotherapy, University Hospital of Wuerzburg, Center of Mental Health, Wuerzburg, Germany

[j] German Centre of Prevention Research in Mental Health, University of Wuerzburg, Germany

Corresponding author*: Dr. rer. nat. Kathrin Schag

University Hospital Tübingen, Department of Psychosomatic Medicine & Psychotherapy

Osianderstraße 5, 72076 Tübingen, Germany

Phone: +49-7071-29-89117

e-mail: kathrin.schag@med.uni-tuebingen.de

S1. Search terms for the literature search in each database

- PubMed: ("Binge-Eating Disorder"[Mesh] OR "Bulimia"[Mesh] OR binge OR bulimi* OR EDNOS OR OSFED OR Night-eating OR “night eating” OR “loss of control eating” OR “loss-of-control eating” OR “eating disorder”) AND (emotion* OR "Emotional Regulation"[Mesh] OR “emotion regulation” OR emotion-related* OR emotion-focused*) AND ("Psychotherapy"[Mesh] OR psychother* OR treatment OR training OR intervention) AND trial
- PsycInfo: (binge OR bulimi* OR EDNOS OR OSFED OR Night-eating OR “night eating” OR “loss of control eating” OR “loss-of-control eating” OR “eating disorder”) AND (emotion* OR “emotion regulation” OR emotion-related* OR emotion-focused*) AND (psychother* OR treatment OR training OR intervention) AND trial
- Cochrane Central Register of Controlled Trials: ((binge or bulimi* or EDNOS or OSFED or Night-eating or night eating or loss of control eating or loss-of-control eating or eating disorder) and (emotion* or emotion regulation or emotion-related* or emotion-focused*) and (psychother* or treatment or training or intervention) and trial) in all text

S2. Summary of the studies investigating emotion-related interventions to reduce binge eating behaviour.

Table S2 a) Studies in adults, table S2 b) Studies in adolescents.

Table S2 a). *Studies in adults.*

Study	Sample (N, diagnosis, BMI)	Emotion-related intervention (type, N, setting, dose)	Control group (type, N, setting, dose)	BE/LOC and general ED pathology	Emotion-related outcomes	Summary of main findings at end of treatment	Summary of main findings at FU
Berking et al. (2022)	*N*=101 adults with BED, BMI < 45	Affect regulation training* (ART, N = 61, 7 group sessions à 180 min, 1 booster session à 90 min, daily individual training)	WC (N=40)	BEF in the last 4 weeks acc. to EDE; EDE total score	ERSQ	ART > WC in improvement of BEF, EDE total score and ERSQ; ERSQ mediator for BEF reduction and EDE total score	Results maintained at 6 months FU
Blood et al. (2020)	*N*=56 adults with BED, BMI = 42.2 (8.7)	DBT-BED skills training (N = 56, 20 group therapy sessions à 120 min)	None	abstinence of BE and BEF in the last 4 weeks acc. to EDE-Q;<br>EDE-Q total score, BES	EES	Significant improvement in BEF, abstinence rate (60%), EDE-Q total score and BES; no significant changes in EES	Results maintained at 1 month FU
Chen et al. (2017)	*N*=109 adults with BED or BN, BMI = 31.5 (8.8)	DBT for non-responders to 4 weeks GSH (N=36, 6 months weekly, 1h individual, 2h skills group, 2h therapist consultation team, 24h phone coaching)	- CBT+ for non-responders to 4 weeks GSH (N=31, 6 months weekly, 1h individual, 2h group, 24h psychiatry on-call)<br>- continued GSH for responders to GSH (N=42, up to 24 sessions within 6 months à 20-30 min)	BE days in the last 4 weeks acc. to EDE;<br>EDE total score	None	DBT = CBT+ < continued GSH in BE days reduction;<br>DBT = CBT+ = continued GSH in EDE total score reduction;	DBT = CBT+ = continued GSH regarding BE days at 6 months and 12 months FU;<br>DBT > CBT+ = continued GSH regarding sustained BE days reduction at 12 months FU

Compare & Tasca (2016)	*N*=126 adults with BED and obesity, BMI = 33.6 (3,6); clinician-rated treatment admission	Combined therapy* of EFGT and dietary counselling (DC) for patients with no primarily emotional urge to BE (N=63, 12 individual sessions à 60 min, 8 group sessions à 30 min in 3 months)	EFGT for patients with primarily emotional urge to BE (N = 55, 20 weekly sessions à 60-90 min in 5 months)	BEF in the last 4 weeks acc. to EDE	None	Combined therapy = EFGT in BEF reduction;<br>BEF and weight covaried and faster reduction of BEF and weight in combined therapy	Results maintained at 6 months FU;<br>rapid change in the mid of treatment related with better outcomes at FU
Compare et al. (2013)	*N*=189 adults with BED, BMI >/= 30; clinician-rated treatment admission	EFT (N = 63, 20 group sessions à 60-90 min)	- DC (N = 63, 12 individual sessions à 60 min, 8 group sessions à 30 min)<br>- combined treatment (N = 63, 20 EFT + 20 DC)	abstinence of BE acc. to EDE	None	Combined treatment > EFT = DC regarding abstinence rate in ITT analysis;<br>Combined treatment = EFT = DC regarding abstinence rate in PP analysis	Combined treatment > EFT > DC regarding abstinence rate at 6 months FU (in ITT and PP analysis)
Duarte et al. (2017)	*N*=20 adults with BED, BMI = 31.9 (6.3)	Mindfulness and compassion-based self-help intervention "CARE" (N = 11, group session à 150 min, 4 weeks mindfulness at home, evaluation session)	WC (N = 9)	BEF in the last 4 weeks acc. to EDE; EDE, BES, BISS, BIAAQ, CFQFC	FFMQ, SCS, CEAS	CARE > WC in improvement of BEF, EDE subscales, BES, BIAAQ, CFQFC, FFMQ nonjudging, CEAS actions;<br>CARE = WC in BISS, FFMQ other subscales, SCS, CEAS engagement	Results maintained at 1 months FU
Fernandez-Aranda et al. (2015)	*N*=38 adults with BN, BMI = 25.5 (5.9)	Serious video game (SVG) addressing emotion regulation and self-control + CBT* (N = 20, 10-12 individual SVG sessions à 90 min, 16 outpatient group CBT sessions à 90 min)	CBT (N = 18, 16 group sessions à 90 min)	remission rate (abstinence in the last 4 weeks at daily diary and psychological improvement in the last 3 months); EDI-2	None	SVG + CBT = CBT regarding remission rate and EDI-2 total score (SVG + CBT higher effect sizes)	None

Glisenti et al. (2021)	*N*=21 adults with BED; BMI N/A	EFT (N = 10, 12 individual therapy sessions à 60 min)	WC (N = 11)	BEF in the last 4 weeks acc. to EDE-Q;<br>BES	None	EFT > WC in reduction of BEF and BES	None
Hill et al. (2011)	*N*=32 adults with full- or subthreshold BN; BMI = 22.6 (SD N/A)	Appetite-focused DBT (DBT-AF, N = 18, 12 individual sessions à 75 min)	WC (N = 14, 8 started treatment 6 weeks delayed)	BEF and purging in the last 4 weeks acc. to EDE;<br>EDE-Q, MACR, IA-E appetite awareness, PEWS	EES, NMRS, IA-E Emotion awareness, PANAS	DBT-AF > WC in reduction of BEF and purging, EDE-Q, MAC-R, PEWS, IA-E appetite awareness, PANAS positive affect;<br>DBT-AF = WC regarding PANAS negative affect, EES, NMRS	None
Juarascio et al. (2017)	*N*=19 adults with BED; BMI = 32.8 (8.9)	Acceptance-based behavioural treatment* (N = 19, 10 group therapy sessions with 8-9 sessions à 90 min and 1-2 sessions à 120 min)	None	BEF in the last 4 weeks acc. to EDE;<br>EDE subscales	DERS, UPPS impulsive behaviour scale – negative urgency, AAQ, FAAQ	significant improvement of BEF, abstinence rate and EDE subscales, UPPS negative urgency, AAQ, FAAQ willingness, DERS nonacceptance;<br>no significant reduction in other DERS subscales and FAAQ acceptance;<br>improvement in DERS associated to EDE reduction	Significant improvement of BEF, abstinence rate and EDE subscales at 3 months FU
Juarascio et al. (2021)	*N*=44 adults with BN; BMI = 25.0 (5.7)	Mindfulness and acceptance-based treatment (MABT, N = 26, 2 individual sessions)	CBT (N = 18, 20 individual sessions)	LOC (sum score of objective and subjective BE) acc. to EDE; EDE total score	DERS, AAQ, DTS	MABT = CBT in improvement of LOC, compensatory behaviours, EDE total score, DERS, AAQ and DTS	Most effects maintained at 6 months FU

Juarascio et al. (2020)	*N*=16 adults with BED or BN; BMI = 32.3 (9.6)	Integrative cognitive-affective therapy (ICAT) + App (N = 16, 21 individual sessions à 50 min + App)	None	LOC (sum score of objective and subjective BE) acc. to EDE; EDE total score	DERS	Significant improvement of LOC, EDE total score, DERS; small compliance on App-based interventions	None
Kelly & Carter (2015)	*N*=41 adults with BED; BMI N/A	Compassion-focused self-help (N = 15, 1 introductory session, 3 weeks individual self-help exercises)	- CBT self-help (N = 13, 1 introductory session, 3 weeks individual self-help exercises)<br>- WC (N = 13)	BE days acc. to self-monitoring per day;<br>EDE-Q total score	SCS	Compassion-focused = CBT > WC in reduction of BE days; Compassion-focused > CBT = WC in reduction of EDE-Q;<br>Compassion-focused > WC in SCS;<br>stronger improvement in EDE-Q for patients with low fear of self-compassion	None
Klein et al. (2012)	*N*=10 adults with sub- or full-threshold BED or BN; BMI N/A	DBT (N = 5 completers, 16 group sessions à 120-150 min)	None	Weekly BEF acc. to diary card;<br>EDI	None	significant reduction regarding BEF and EDI total score;<br>high attrition rate	None
Klein et al. (2013)	*N*=36 adults with sub- or full-threshold BED or BN; BMI N/A	DBT (N = 8 completers, 15 group sessions à 150 min)	Self-monitoring of urges, behaviours and emotions by diary card (N = 12 completers, 15 individual sessions à 15 min and self-monitoring)	Weekly BEF acc. to diary card; change from full- to subthreshold diagnosis;<br>EDI	None	DBT > self-monitoring in diagnosis change;<br>significant reduction of BEF and EDI subscales in DBT, in self-monitoring only EDI subscales Bulimia and Interoceptive Awareness;<br>high attrition rate	None
Kristeller et al. (2014)	*N*=150 adults with sub- or full-threshold	Mindfulness-based Eating Awareness Training (MB-EAT, N = 53, 12 group	- Psychoeducation (PED, N = 50, 12 group sessions à 90-120 min)<br>- WC (N = 47)	BE days in the last 4 weeks acc. to EDE;	None	MB-EAT = PED > WC regarding abstinence rate, improvement of BE days, BES, TFEQ, PFS, ESES;	At 4 months FU MB-EAT > PED > WC regarding abstinence rate and BE size;

	BED; BMI = 40.3 (Range 26-78)	sessions à 90-120 min and homework)		BEF and size acc. to weekly self-monitoring;<br>BES, TFEQ, PFS, ESES		MB-EAT > PED regarding TFEQ hunger, TFEQ inhibition and PFS food available subscale;<br>Amount of mindfulness training associated to improvement in BES, TFEQ, PFS	MB-EAT = PED > WC regarding other outcomes
Kristeller & Hallett (1999)	*N*=21 adults with BED; BMI >/= 27 BMI = 40.3 (Range 28-52)	Mindfulness-based meditation (N = 18, 7 group sessions)	None	BEF, portion size and sense of control weekly assessed in self-reports; BES	Mindful eating weekly assessed in self-reports	Significant improvements in BEF, portion size, BES, sense of eating control, mindful eating; amount of meditation associated to BES reduction,<br>sense of eating control, sense of mindfulness and awareness of satiety cues associated to BE reduction	None
Lammers et al. (2020)	*N*=74 adults with BED and above average emotional eating; BMI = 39.9 (5.6)	DBT-BED (N = 41, 20 group sessions à 120 min)	CBT+ (N = 33, 20 group sessions à 75 min, 6 supportive meetings à 90 min)	BEF in the last 4 weeks acc. to EDE-Q;<br>EDE-Q total score	DEBQ subscale emotional eating, EDI-3 subscale emotion dysregulation	CBT+ > DBT-BED in reduction of BEF;<br>DBT-BED = CBT+ in reduction of EDE-Q total score, DEBQ emotional eating, EDI-3 emotion dysregulation	At 6 months FU DBT-BED = CBT+ in reduction of BEF, DEBQ emotional eating, EDI-3 emotion dysregulation;<br>CBT+ > DBT-BED in reduction of EDE-Q total score
Lammers et al. (2022)	*N*=175 adults with sub- or full-threshold BED; BMI = 42.8 (7.4)	DBT-BED (N = 42, 20 group sessions à 120 min)	CBT+ (N = 133, 20 group sessions à 75 min, 6 supportive meetings à 90 min)	BEF in the last 4 weeks acc. to EDE-Q;<br>EDE-Q total score	DEBQ subscale emotional eating, EDI-3 subscale	DBT-BED = CBT+ in reduction of BEF;<br>CBT+ > DBT-BED in reduction of EDE-Q total score, DEBQ emotional	At 6 months FU DBT-BED = CBT+ in reduction of BEF, EDE-Q total score, DEBQ emotional eating;

					emotion dysregulation	eating, EDI-3 emotion dysregulation	CBT+ > DBT-BED in reduction of EDI-3 emotion dysregulation
Lavender et al. (2012)	*N*=74 adults with BN or EDNOS (BED); BMI = 24.9 (6.7)	Emotional & social mind training* (ESM, N = 35, 4 individual sessions, 12 group sessions, 1 FU group session, each 60-90 min)	CBT (N = 35, 4 individual sessions, 12 group sessions, 1 FU group session, each 60-90 min)	BEF and purging in the last 4 weeks acc. to EDE; EDE total score	Beliefs about Emotions Scale, DTS	ESM = CBT in improvements of BEF, purging, EDE total score, Beliefs about emotions scale, DTS; Diagnosis, Beliefs about emotions and DTS no moderators of change in EDE total score	Results maintained at 6 months FU
Masson et al. (2013)	*N*=60 adults with BED; BMI =37.1 (8.8)	DBT-BED GSH (N = 30, 1 individual orientation session à 45 min, 6 individual biweekly phone calls à 20 min)	WC (N = 30)	BEF in the last 4 weeks acc. to EDE-Q, remission rate acc. EDE diagnostic items; EDE-Q total score, EDQLS	DERS	DBT-BED GSH > WC in improvement of BEF, remission rate, EDE-Q total score, EDQLS and DERS	Significant improvement in DBT-BED GSH regarding all outcomes at 6 months FU
McIntosh et al. (2016)	*N*=112 adults with transdiagnostic BE diagnosis; BMI = 30.0 (7.9)	Schema therapy (N = 38, 6 months weekly individual sessions, 6 months monthly sessions)	- CBT (N = 38)<br>- appetite-focused CBT (CBT-A, N = 36)<br>- dose of both treatments like Schema therapy	BEF in the last 4 weeks and purging acc. to EDE-12; EDE-12 total score	None	Schema = CBT = CBT-A in reduction of BEF, purging, abstinence, EDE-12 total score;<br>BED > BN in reduction of BEF	Results maintained at 12 months FU;<br>BED = BN in reduction of BEF at 12 months FU
Peterson et al. (2020)	*N*=112 adults with BED; BMI = 33.7 (8.4)	ICAT (N = 56, 21 individual sessions à 50 min, 8 twice a week, 13 weekly)	CBT GSH (N = 56, 1 individual session à 60 min, 9 individual sessions à 30min, session 1-4 weekly, session 5-10 biweekly)	BEF in the last 4 weeks acc. to EDE, BEF probability within 7 days; EDE total score	DERS	ICAT = CBT GSH in improvement of BEF, abstinence rate, EDE total score, BEF probability and DERS	ICAT = CBT in improvement of BEF, abstinence rate and DERS at 6 months FU

Petersson et al. (2022)	*N*=40 adults (AN N=6, BN N=3, BED N=6, OSFED N=25); BMI = 23.5 (5.8)	PED “Affect School” + TAU (N = 21, 8 group sessions à 120 min)	TAU with focus on CBT (N = 19)	BEF in the last two weeks acc. to self-report;<br>EDE-Q total score	DERS-36, TAS-20	TAU > Affect School + TAU in BEF reduction (descriptive results);<br>Affect School + TAU = TAU in improvement in EDE-Q total score, DERS-36, TAS-20	Affect School + TAU > TAU in improvement of all outcomes at 6 months and 12 months FU, besides TAS-20 (only at 6, not 12 months FU)
Preuss et al. (2017)	*N*=69 patients with obesity (N=23 BED subgroup); BMI = 33.1 (3.2)	treatment to increase inhibitory control and emotion regulation “ImpulsE”* (N = 41, 10 group sessions à 100 min, 1 booster session, inhibitory control training)	TAU (CBT) (N = 28, 10 group sessions à 100min, 1 booster session)	BE days in the last 4 weeks and disinhibited overeating acc. to EDE-Q;<br>EDE-Q total score	UPPS Impulsive Behaviour Scale – negative urgency	ImpulsE = TAU (CBT) in reduction of BE days (BED subgroup) and disinhibited overeating (total sample), EDE-Q total score, UPPS negative urgency	Results maintained at 3 months FU;<br>BED > patients with obesity regarding treatment benefit at 3 months FU
Robinson (2013)	*N*=16 adults with BED; BMI = 31.2 (9.4)	Integrative response therapy* (N = 16, 10 group sessions à 60 min)	None	BE days in the last 4 weeks acc. to EDE-Q;<br>EDE-Q total score	EES	significant improvement of BE days, EDE-Q total score, EES, 54% abstinence rate	Results maintained at 3 months FU, 67% abstinence rate
Safer et al. (2001)	*N*=31 adults with BN; BMI = 23.7 (5.6)	DBT (N = 14, 20 individual sessions à 50 min	WC (N = 15)	BEF and purging in the last 4 weeks acc. to EDE	EES, NMRS, PANAS	DBT > WC in reduction of BEF, purging; 28.6% vs. 0% abstinence rate;<br>DBT = WC in improvement of EES, NMRS, PANAS	None
Safer et al. (2010)	*N*=101 adults with BED; BMI = 35.8 (9.4)	DBT-BED (N = 50, 20 group sessions à 120 min)	Active comparison with supportive group treatment (ACGT, N = 51, 20 group sessions à 120 min)	BE days in the last 4 weeks and abstinence rate acc. to EDE;<br>EDE subscales	EES, NMRS, DERS, PANAS	DBT > ACGT in reduction of BE days, 64% vs. 36% abstinence rate, EDE subscales, EES;<br>no changes in NMRS, DERS, PANAS	DBT = ACGT at 3, 6 and 12 months FU in reduction of BEF, abstinence rate;<br>DBT > ACGT in reduction of EDE restraint and weight

							concern at 12 months FU;<br>ACGT > DBT regarding DERS, PANAS negative emotions at 12 months FU
Tanis et al. (2023)	*N*= 92 adults with BED, BN or UFED (BE); BMI = 32.4 (7.0)	Emotion-focused implementation intentions (N = 18, 3 weekly sessions à 45 min)	- behaviour-focused implementation intentions (N = 20)<br>- goal setting (N = 26)<br>- same dose of all three treatments	BEF by online food diary 3 times a day per week over four weeks,<br>BEF in the last 3 weeks acc. to EDE-Q;<br>EDE-Q (21 days) total score	None	both implementation intention conditions > goal setting in reduction of BEF (EDE-Q and food diary), emotion-focused = behaviour-focused implementation intention; no effect on EDE-Q total score	both implementation intention conditions > goal setting in reduction of BEF at 1, 3 and 6 months FU; reduction of EDE-Q total score at 1, 3 and 6 months in all conditions
Telch et al. (2000)	*N*=11 adults with BED; BMI = 37.3 (8.9)	DBT-BED (N = 11, 20 group sessions à 120 min)	None	BEF in the last 4 weeks acc. to EDE; EDE subscales, BES	EES, NMRS, PANAS	improvement of BEF (strong effect), 82% abstinence rate, EDE subscales, BES, EES, NMRS, PANAS positive emotions	Abstinence rates maintained at 3 months (80%) and 6 months (70%) FU
Telch et al. (2001)	*N*=44 adults with BED; BMI =36.4 (6.6)	DBT-BED (N = 22, 20 group sessions à 120 min)	WC (N = 22)	BEF in the last 4 weeks acc. to EDE; EDE subscales, BES	EES, NMRS, PANAS	DBT-BED > WC in reduction of BEF, BES and EDE subscales (besides restraint), EES anger, 89% vs. 12% abstinence rate; no change in other EES subscales, NMRS, PANAS (PP analyses)	In DBT-BED abstinence rates reduced to 67% at 3 months FU and 56% at 6 months FU (PP analyses)
Wnuk et al. (2015)	N = 14 adults with BED, BN or EDNOS; BMI = 28.7 (13.6)	EFT (N = 12, 16 group sessions)	None	BEF and purging in the last 4 weeks acc. to the Past 28	DERS; emotion self-efficacy per session	Improvement of BEF, symptom self-efficacy, EDI subscales Bulimia, Interoceptive Awareness,	None

				Days Eating and Symptoms Interview of DSM-IV; symptom self-efficacy per session, EDI subscales		body dissatisfaction and Ineffectiveness, no changes in purging; improvement in DERS and emotion self-efficacy	
Wonderlich et al. (2014)	*N*=80 adults with sub- or full-threshold BN; BMI =23.5 (5.5)	ICAT (N = 40, 21 individual sessions à 50 min)	CBT+ (N = 40, 21 individual sessions à 50 min)	BEF and purging in the last 4 weeks acc. to EDE; EDE total score	DERS	ICAT = CBT-E in improvement of BEF, purging, abstinence rate (37.5 vs. 22.5%), EDE total score, DERS	ICAT = CBT-E in all outcomes at 4 months FU

Table S2 b). Studies in adolescents.

Study	Sample (N, diagnosis)	Emotion-related intervention (type, N, setting, dose)	Control group (type, N, setting, dose)	BE/LOC and general ED pathology	Emotion-related outcomes	Summary of main findings at end of treatment	Summary of main findings at FU
Fischer & Peterson (2015)	*N*=10 adolescents with EDNOS (BE), BMI = 34.5 (SD N/A)	DBT (N = 10, 24 individual sessions, 24 group sessions)	None	BEF and purging in the last 4 weeks acc. to EDE; EDE total score	Self-harm	Significant reduction in BEF, purging, EDE total score, self-harm	Further improvement of all outcomes at 6 months FU
Kamody et al. (2019)	*N*=30 adolescents with emotional overeating; BMI N/A	DBT (N = 15, 10 group sessions à 60 min)	None	BEF in the last 4 weeks acc. to EDE-Q (self- and parent-report)	EES-Children (self- and parent-report)	Reduction of BEF and EES-Children in self- and parent-report (descriptive results); 50% attrition rate	Further improvement of all outcomes at 3 months FU

Murray et al. (2015)	*N*=35 adolescents with BN; BMI = 26.3 (2.3)	Family-based therapy and DBT* (N = 35, combination of individual, family and parent-only treatment up to 6 days a week for 3–10 hours per day, M= 77 days)	None	BEF and purging in the last 4 weeks acc. to EDE-Q;<br>EDE-Q total score	DERS	Significant reduction in BEF, purging, EDE-Q total score and DERS emotion regulation strategies (not in total score)	None
Salbach et al. (2007)	*N*=31 inpatient adolescents (N=17 AN-R, N=6 AN-BP, N=8 BN); BMI N/A	DBT (N = 31, N = 14 BE subgroup, inpatient treatment with individual and group sessions, flexible dose)	None	Severity of BE in calories and purging acc. to SIAB;<br>SIAB, EDI-2	None	Significant reduction of BE calories, purging (BE subgroup);<br>fasting, excessive exercise and EDI-2 subscales (total sample)	None
Salbach-Andrae et al. (2009)	*N*=50 adolescents (N=26 AN-R, N=11 AN-BP, N=13 BN); BMI N/A	DBT (N = 16, N = 7 BE subgroup, 25 individual sessions à 50 min, 25 group skill trainings à 100 min)	- CBT (N = 19, N = 11 BE subgroup, dose like DBT)<br>- WC (N = 15, N = 6 BE subgroup, individual supportive sessions every second week à 30 min)	BEF acc. to SIAB Likert rating and purging;<br>SIAB abstinence rate from ED diagnosis and total score	None	DBT = CBT > WC in reduction of BEF and purging (BE subgroup);<br>abstinence rate (62,5% vs. 57,9% vs. 0%) and reduction of SIAB total score (total sample)	None

Note. Studies are presented in alphabetical order.
AAQ, Acceptance and Action Questionnaire; ACGT, Active comparison with supportive group treatment; AN-BP, Anorexia Nervosa binge-purge subtype; AN-R, Anorexia Nervosa restrictive subtype; ART, Affect regulation training; BE, Binge Eating; BED, Binge Eating Disorder; BEF, Binge Eating Frequency; BES, Binge Eating Scale; BIAAQ, Body Image Acceptance and Action Questionnaire; BISS, Body Image Shame Scale; BMI, Body Mass Index (M and SD, if available); BN, Bulimia Nervosa; CBT, Cognitive behavioural therapy; CBT+, enhanced CBT for BE according to Fairburn; CEAS, Compassionate Engagement and Action Scales; CFQFC, Cognitive Fusion Questionnaire for food craving; DBT, Dialectical behaviour therapy; DBT-AF, Appetite-focused DBT; DC, Dietary counselling; DEBQ, Dutch Eating Behaviour Questionnaire; DERS, Difficulties in emotion regulation scale; DTS, Distress Tolerance Scale; EDE, Eating Disorder Examination; EDE-Q, Eating Disorder Examination Questionnaire; EDI, Eating Disorder Inventory; EDNOS, Eating Disorders Not Otherwise Specified ; EDQLS, Eating Disorders Quality of Life Scale; EES, Emotional Eating Scale; EFGT, Emotion-focused group therapy; EFT, Emotion-focused therapy; ERSQ, Emotion regulation skills questionnaire; ESES, Eating Self-Efficacy Scale; ESM, Emotional & social mind training; FAAQ, food craving acceptance and action

questionnaire; FFMQ, Five-Facet Mindfulness Questionnaire; FU, Follow-up; GSH, Guided self-help; IA-E, Interoceptive Awareness Scale; ICAT, Integrative cognitive-affective therapy; ITT, Intention-to-treat analysis; LOC, Loss of control eating; MACR, Mizes Anorectic Cognitions Scale-Revised; MABT, Mindfulness and acceptance based treatment; MB-EAT, Mindfulness-based Eating Awareness Training; NMRS, Negative Mood Regulation Scale; PANAS, Positive And Negative Affective Scales; PED, Psychoeducation; PEWS, Preoccupation with Eating Weight and Shape Scale; PFS, Power of Food Scale; PP, Per Protocol Analysis; SCS, Self-Compassion Scale; SIAB, Structured Interview for Anorexia Nervosa and Bulimia Nervosa; SVG, serious video game; TAS-20, Toronto Alexythymia Scale; TAU, Treatment as usual; TFEQ, Three-Factor Eating Questionnaire; WC, Waitlist Control group;
* combined treatments

S3. Statistical values of meta-analytical subgroup analyses

Table S3 a). *Subgroup-analysis of different diagnoses.*

Diagnosis	k	SMD	SE	t	df	p
BED	13	1.6437	0.1991	8.2563	19	<.001
BN	2	1.3259	0.4858	2.7295	19	<.05
Mixed	7	1.4194	0.2896	4.9012	19	<.001
Other	1	0.4669	0.8246	0.5662	19	0.58

Note. Test for between-group differences: Q = 2.17, df = 3, p = .52. BED = Binge Eating Disorder, BN = Bulimia Nervosa, df = degrees of freedom, SE = standard error, SMD = standardized mean difference.

Table S3 b). *Dichotomous subgroup-analysis of different diagnoses (BED vs. all other diagnoses).*

Diagnosis	k	SMD	SE	t	df	p
BED	13	1.6442	0.1949	8.4347	21	<.001
Other	10	1.3146	0.2336	5.6279	21	<.001

Note. Test for between-group differences: Q = .81, df = 1, p = .25. BED = Binge Eating Disorder, df = degrees of freedom, SE = standard error, SMD = standardized mean difference.

Table S3 c). *Subgroup-analysis of different treatments.*

Treatment	k	SMD	SE	t	Df	p
DBT	10	1.6352	0.2447	6.6834	19	<.001
CBTE	3	1.4453	0.4344	3.3269	19	<.01
Mixed	4	1.5606	0.3803	4.1032	19	<.001
Other	5	1.4453	0.3051	4.3135	19	<.001

*Note***.** Test for between-group differences: Q =.62, df = 3, p = .67. CBTE = Cognitive Behavioral Therapy emotion-focused, DBT = Dialectic Behavioral Therapy, df = degrees of freedom, SE = standard error, SMD = standardized mean difference.

Table S3 d). *Dichotomous subgroup-analysis of different treatments (DBT vs. all other treatments).*

Diagnosis	k	SMD	SE	t	df	p
DBT	10	1.6340	0.2344	6.9711	21	<.001
Other	13	1.4189	0.1998	7.1025	21	<.001

Note. Test for between-group differences: Q = .23, df = 1, p = .53. DBT = Dialectic Behavioral Therapy, df = degrees of freedom, SE = standard error, SMD = standardized mean difference.

Table S3 e). *Subgroup-analysis of different time frames in outcome variables.*

Outcome	k	SMD	SE	t	df	p
28 days	18	1.5255	0.1688	9.0362	20	<.0001
7 days	4	1.6460	0.4181	3.9369	20	<.001
not reported	1	0.7437	0.7562	0.9836	20	.3371

Note. Test for between-outcome differences: Q = 1.17, df = 2, p = .56. df = degrees of freedom, SE = standard error, SMD = standardized mean difference.

Table S3 f). *Subgroup-analysis of different instruments used in outcome variables.*

Outcome	k	SMD	SE	t	df	p
EDE	20	1.5117	0.1612	9.3762	20	<.0001
other	2	1.9145	0.5632	3.3993	20	<.05
not reported	1	0.7437	0.7492	0.9927	20	.3327

Note. Test for between-outcome differences: Q = 3.30, df = 2, p = .19. df = degrees of freedom, SE = standard error, SMD = standardized mean difference.

S4. Overview on used emotion regulation instruments in the identified studies

Emotion regulation instrument	Target construct	Number of items and subscales	Studies using this instrument
Acceptance and Action Questionnaire – II (AAQ- II)<br>(Bond et al. 2011)	Psychological inflexibility, as defined by ACT theory	7 items	Juarascio et al. (2017);<br>Juarascio et al. (2021)
Beliefs about emotions scale (BES)<br>(Rimes & Chalder, 2010)	Individuals' beliefs about the (un)acceptability of experiencing and expressing emotions	12 items	Lavender et al. (2012)
Compassionate Engagement and Action Scales (CEAS)<br>(Gilbert et al. 2017)	Compassionate engagement and action	39 items;<br>3 subscales: (a) from others, (b) for others (b) and (c) self- compassion	Duarte et al. (2017)
Deliberate Self-Harm Questionnaire (DSHQ)<br>(Gratz, 2001)	Behaviourally based assessment of deliberate self-harm without suicide intend	17 items	Fischer & Peterson (2015)
Difficulties in emotion regulation scale (DERS)<br>(Gratz & Roemer, 2004)	Emotion regulation as individuals' ability to understand, accept and manage emotional states	36 items;<br>6 subscales: (a) awareness and understanding of emotions, (b) non-acceptance of emotions, (c) difficulties to engage in goal-directed behaviours, (d) non-acceptance of emotional states, (e) impulse control difficulties, (f) limited access to emotion regulation strategies and (g) lack of emotional clarity	Juarascio et al. (2017);<br>Juarascio et al. (2021);<br>Juarascio et al. (2020);<br>Masson et al. (2013);<br>Murray et al. (2015);<br>Peterson et al. (2020);<br>Petersson et al. (2022);<br>Safer et al. (2010);<br>Wnuk et al. (2015);<br>Wonderlich et al. (2014)
Dutch Eating Behaviour Questionnaire (DEBQ) – subscale emotional eating<br>(van Strien et al. 1986)	Frequency of eating due to unpleasant emotions	13 items	Lammers et al. (2020);<br>Lammers et al. (2022)

Distress Tolerance Scale (DTS)<br>(Simons & Gaher, 2005)	Extent to which individuals believe they have the ability to experience and withstand distressing emotional states	15 items	Juarascio et al. (2021);<br>Lavender et al. (2012)
Eating Disorder Inventory 3rd edition (EDI-3), subscale emotional dysregulation<br>(Garner, 2004)	Poor impulse control and mood intolerance	8 items	Lammers et al. (2020);<br>Lammers et al. (2022)
Emotional Eating Scale (EES)<br>(Arnow, Kenardy & Argas, 1995)	Intensity of relationship between (disordered) desire to eat and mood	25 items;<br>3 subscales: (a) anger/ frustration, (b) anxiety, (c) depression	Blood et al. (2020);<br>Hill et al. (2011);<br>Kamody et al. (2019) (EES-Children; self- and parent-report);<br>Robinson (2013);<br>Safer et al. (2001);<br>Safer et al. (2010);<br>Telch et al. (2000);<br>Telch et al. (2001)
Emotion regulation skills questionnaire (ERSQ)<br>(Grant, Salsman & Berking, 2018)	Extend to which individual can successfully apply skills to regulate emotions	27 items;<br>9 subscales: (a) awareness of emotions, (b) interpretation of sensations related to emotions, (c) clarity, (d) understanding, (e) modification, (f) acceptance, (g) tolerance, (h) readiness to confront distressing situations when necessary to attain personal relevant goals, (i) self-support)	Berking et al. (2022)
Food craving acceptance and action questionnaire (FAAQ)<br>(Juarascio, Forman, Timko, Butryn & Goodwin, 2011)	Acceptance to internal feelings specifically related to eating behaviour	10 items;<br>2 subscales: (a) ability to regulate eating despite craving and (b) desire to maintain control over internal eating thoughts	Juarascio et al. (2017)

Five-Facet Mindfulness Questionnaire (FFMQ)<br>(Baer, Hopkins, Krietemeyer & Toney, 2006)	Mindfulness	37 items;<br>5 subscales: (a) nonreactivity to inner experiences, (b) observing thoughts/feelings, (c) acting with awareness, (d) describing with words, (e) nonjudging of experience	Duarte et al. (2017)
Interoceptive Awareness Scale – Expanded (IA-E)<br>(Craighead & Niemeier, 2002)	Appetite awareness and emotion awareness	18 items	Hill et al. (2011)
Negative Mood Regulation Scale (NMRS)<br>(Catanzaro & Mearns, 1990)	Extend to which individuals believe negative mood can be regulated by thoughts or behaviours	30 items	Hill et al. (2011);<br>Safer et al. (2001);<br>Safer et al. (2010);<br>Telch et al. (2000);<br>Telch et al. (2001)
Positive And Negative Affective Scales (PANAS)<br>(Watson, Clark & Tellegen, 1988)	Extend to which individuals experience affect	20 items;<br>2 subscales: (a) positive affect and (b) negative affect	Hill et al. (2011);<br>Safer et al. (2001);<br>Safer et al. (2010);<br>Telch et al. (2000);<br>Telch et al. (2001)
Self-Compassion Scale (SCS)<br>(Neff, 2003)	Self-compassion	26 items;<br>3 subscales: (a) self-kindness, (b) common humanity, (c) mindfulness	Duarte et al. (2017);<br>Kelly & Carter (2015)
Toronto Alexithymia Scale (TAS-20)<br>(Bagby, Taylor & Parker, 1993)	Difficulties in identifying and describing emotions	20 items	Petersson et al. (2022)
UPPS impulsive behaviour scale, subscale negative urgency<br>(Whiteside et al. 2005; Whiteside & Lynam, 2001)	Extend to which impulsive behaviour is shown under negative mood	12 items	Juarascio et al. (2017);<br>Preuss et al. (2017)

Note. Instruments are presented in alphabetical order.

References in table S4 that are not included in the main text:

Arnow, B., Kenardy, J., & Agras, W. S. (1995). The emotional eating scale: The development of a measure to assess coping with negative affect by eating. *International Journal of Eating Disorders*, *18*(1), 79–90. https://doi.org/10.1002/1098-108X(199507)18:1<79::AID-EAT2260180109>3.0.CO;2-V

Baer, R. A., Smith, G. T., Hopkins, J., Krietemeyer, J., & Toney, L. (2006). Using self-report assessment methods to explore facets of mindfulness. *Assessment*, *13*(1), 27–45. https://doi.org/10.1177/1073191105283504

Bagby, R. M., Taylor, G. J., & Parker, J. D. A. (1994). The twenty-item Toronto Alexithymia scale-II. Convergent, discriminant, and concurrent validity. *Journal of Psychosomatic Research*, *38*(1), 33–40. https://doi.org/10.1016/0022-3999(94)90006-X

Bond, F. W., Hayes, S. C., Baer, R. A., Carpenter, K. M., Guenole, N., Orcutt, H. K., Waltz, T., & Zettle, R. D. (2011). Preliminary Psychometric Properties of the Acceptance and Action Questionnaire-II: A Revised Measure of Psychological Inflexibility and Experiential Avoidance. *Behavior Therapy*. https://doi.org/10.1016/j.beth.2011.03.007

Catarizaro, S. J., & Mearns, J. (1990). Measuring Generalized Expectancies for Negative Mood Regulation: Initial Scale Development and Implications. In *Journal of Personality Assessment* (Vol. 54, Issues 3–4, pp. 546–563). https://doi.org/10.1080/00223891.1990.9674019

Craighead, L. W., & Niemeier, H. M. (2002). *The Interoceptive Awareness Scale-Expanded*. Boulder, CO: University of Colorado.

Gilbert, P., Catarino, F., Duarte, C., Matos, M., Kolts, R., Stubbs, J., Ceresatto, L., Duarte, J., Pinto-Gouveia, J., & Basran, J. (2017). The development of compassionate engagement and action scales for self and others. *Journal of Compassionate Health Care*, *4*(1), 1–24. https://doi.org/10.1186/s40639-017-0033-3

Grant, M., Salsman, N. L., & Berking, M. (2018). The assessment of successful emotion regulation skills use: Development and validation of an English version of the Emotion Regulation Skills Questionnaire. *PLoS ONE*, *13*(10), 27–28. https://doi.org/10.1371/journal.pone.0205095

Gratz, K. L. (2001). Measurement of deliberate self-harm: Preliminary data on the deliberate self-harm inventory. *Journal of Psychopathology and Behavioral Assessment*, *23*(4), 253–263. https://doi.org/10.1023/A:1012779403943

Gratz, K. L., & Roemer, L. (2004). Multidimensional Assessment of Emotion Regulation and Dysregulation: Development, Factor Structure, and Initial Validation of the Difficulties in Emotion Regulation Scale. *Journal of Psychopathology and Behavioral Assessment*, *26*(1), 41–54. https://doi.org/10.1023/B:JOBA.0000007455.08539.94

Juarascio, A., Forman, E., Timko, C. A., Butryn, M., & Goodwin, C. (2011). The development and validation of the food craving acceptance and action questionnaire (FAAQ). *Eating Behaviors*, *12*(3), 182–187. https://doi.org/10.1016/j.eatbeh.2011.04.008

Murray, T. C., Rodgers, W. M., & Fraser, S. N. (2009). Examining implementation intentions in an exercise intervention: The effects on adherence and self-efficacy in a naturalistic setting. *Journal of Applied Social Psychology*, *39*(10), 2303–2320. https://doi.org/10.1111/j.1559-1816.2009.00527.x

Neff, K. (2003). The Relational Compassion Scale: Development and Validation of a new self rated Scale for the Assessment of Self Other Compassion. *Self and Identity*, *2*(3), 223–250. https://doi.org/10.1080/15298860390209035

Rimes, K. A., & Chalder, T. (2010). The Beliefs about Emotions Scale: Validity, reliability and sensitivity to change. *Journal of Psychosomatic Research*, *68*(3), 285–292. https://doi.org/10.1016/j.jpsychores.2009.09.014

Simons, J. S., & Gaher, R. M. (2005). The distress tolerance scale: Development and validation of a self-report measure. *Motivation and Emotion*, *29*(2), 83–102. https://doi.org/10.1007/s11031-005-7955-3

van Strien, T., Frijters, J. E. R., Bergers, G. P. A., & Defares, P. B. (1986). The Dutch Eating Behavior Questionnaire (DEBQ) for assessment of restrained, emotional, and external eating behavior. *International Journal of Eating Disorders*, *5*(2), 295–315. https://doi.org/10.1002/1098-108X(198602)5:2<295::AID-EAT2260050209>3.0.CO;2-T

Watson, D., Clark, L. A., & Tellegen, A. (1988). Development and Validation of Brief Measures of Positive and Negative Affect: The PANAS Scales. *Journal of Personality and Social Psychology*, *54*(6), 1063–1070. https://doi.org/10.1037/0022-3514.54.6.1063

Whiteside, S. P., Lynam, D. R., Miller, J. D., & Reynolds, S. K. (2005). Validation of the UPPS impulsive behaviour scale: A four-factor model of impulsivity. *European Journal of Personality*, *19*(7), 559–574. https://doi.org/10.1002/per.556
